# Metabolic Reprogramming in Colorectal Cancer: The Impact of Fatty Acid Metabolism

**DOI:** 10.1155/humu/9567214

**Published:** 2025-09-22

**Authors:** Zirui Zhuang, Yu Chen, Yizhou Yao, Xinguo Zhu

**Affiliations:** Department of General Surgery, The First Affiliated Hospital of Soochow University, Suzhou, China

**Keywords:** cancer treatment, colorectal cancer, diagnostic marker, fatty acid metabolism, immune microenvironment

## Abstract

Colorectal cancer (CRC) ranks among the most common malignant tumors worldwide, with the metabolism of fatty acids being crucial for its development and progression. Altered fatty acid metabolism is a well-established metabolic characteristic of malignant tumors, including CRC. A complex and reciprocal relationship exists between fatty acid metabolism and CRC. On one side, the emergence and advancement of CRC can trigger a reprogramming of fatty acid metabolism. To fulfill the requirements of rapid cell division and survival, cancer cells increase both the synthesis and uptake of fatty acids while also suppressing their oxidation. Conversely, modifications in fatty acid metabolism can affect CRC, as abnormal fatty acid byproducts may activate signaling pathways that foster tumor cell proliferation, thus enhancing tumor progression. Understanding the interplay between fatty acid metabolism and the early stages and advancement of CRC, in conjunction with its relationship with the tumor microenvironment, is a vital area for future investigation. This article reviews the most recent discoveries concerning the impact of fatty acid metabolism on CRC progression, with the objective of supplying a solid theoretical framework and innovative perspectives for additional research and treatment of this condition.

## 1. Introduction

Worldwide, colorectal cancer (CRC) holds the third position in incidence rates, representing approximately 10% of all cancer cases, and is the second leading cause of cancer-related deaths [1]. The morbidity and mortality rates associated with CRC exhibit significant regional variations. In developed countries, such as the United States and certain European nations, the incidence of CRC is elevated due to the Westernization of lifestyles, characterized by high-calorie and high-fat diets, coupled with decreased physical activity. Conversely, in developing countries, the incidence of CRC is also on the rise, driven by economic growth and corresponding lifestyle changes [2]. Furthermore, there are slight differences in incidence rates between genders, with males experiencing a marginally higher overall incidence than females. Age is a significant factor influencing the incidence of CRC, as the majority of diagnoses occur in individuals aged 50 or older; however, there has been a notable increase in cases among younger patients in recent years. Anatomically, CRC can manifest in various segments of the colon and rectum, with the rectum and sigmoid colon being the most commonly affected sites [3]. Moreover, several risk factors are associated with the onset of CRC, such as a family history of the disease, inflammatory bowel conditions, obesity, and diabetes, all of which increase a person's chances of developing this illness.

**Figure 1:**
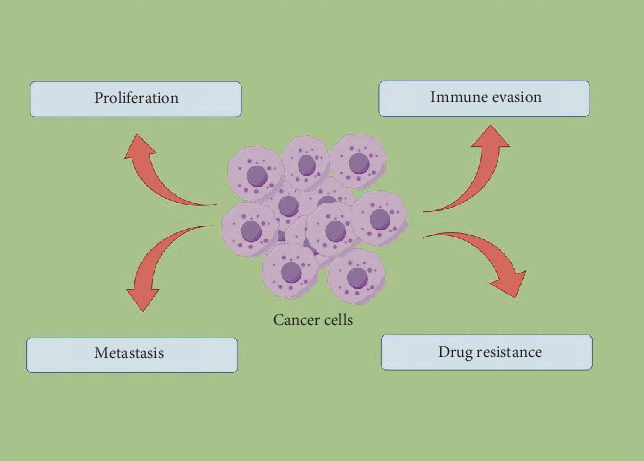
The role of fatty acid metabolism in tumors.

Altered fatty acid metabolism has been thoroughly investigated and validated in CRC. Studies show that the metabolic reprogramming of fatty acids is a key characteristic of cancer cells, playing a crucial role in the onset and advancement of CRC [4]. Fatty acid metabolism encompasses multiple processes, including the uptake, desaturation, oxidation, and synthesis of fatty acids. Dysregulation in these processes can lead to cancer cell proliferation, immune evasion, metastasis, and drug resistance [5] (Figure 1). Fatty acid synthase (FASN) is notably overexpressed in CRC, which is closely associated with the invasive and metastatic abilities of cancer cells. By influencing the Wnt signaling pathway, FASN promotes the advancement of CRC [6]. Irregularities in the fatty acid oxidation (FAO) process are identified as critical factors for the survival and proliferation of CRC cells [7]. The research additionally uncovered that genetic variations linked to fatty acid metabolism show a significant relationship with the likelihood of developing CRC. For example, single-nucleotide polymorphisms (SNPs) within genes that play a role in fatty acid metabolism could impact serum lipid levels, which in turn may indirectly influence the onset of CRC [8]. Moreover, long noncoding RNAs (lncRNAs) associated with fatty acid metabolism are viewed as promising biomarkers for forecasting overall survival in patients with CRC [9]. In terms of treatment, inhibitors targeting fatty acid metabolism are considered potential therapeutic agents for CRC. By inhibiting fatty acid synthesis or oxidation, the growth and survival of cancer cells can be effectively suppressed. Additionally, studies have indicated that the interaction between fatty acid metabolism and the gut microbiota may significantly influence the development of CRC, suggesting that regulating levels of short-chain fatty acids (SCFAs) could represent a novel therapeutic strategy [10]. In conclusion, abnormal fatty acid metabolism is closely associated with the incidence of CRC. An in-depth investigation of its mechanisms will not only enhance our understanding of the pathophysiology of CRC but may also provide critical insights for the development of new diagnostic and therapeutic strategies.

## 2. Fatty Acid Metabolism and CRC

### 2.1. Development of Fatty Acid Metabolism Biomarkers in CRC

Fatty acid metabolism encompasses the synthesis, breakdown, and transformation of fatty acids within the body. It is a crucial component of cellular energy metabolism, not only providing energy to cells but also participating in various physiological functions, such as cell signaling, immune response regulation, and the maintenance of homeostasis. In the context of tumor progression, the reprogramming of fatty acid metabolism is recognized as a critical mechanism through which cancer cells adapt to hostile environments, enhance proliferation, and facilitate metastasis. Consequently, a comprehensive investigation into the biomarkers linked to fatty acid metabolism will improve our comprehension of the processes by which fatty acid metabolism promotes the aggressive proliferation of cancer cells. Recent research has indicated that reducing FUT2 levels leads to a decline in glucose absorption and de novo synthesis of fatty acids, ultimately hindering the growth and spread of CRC cells. On a mechanistic level, FUT2 promotes the movement of YAP1 into the nucleus and helps stabilize mSREBP-1 via fucosylation, which in turn boosts de novo fatty acid synthesis in CRC cells [11]. Moreover, SDHC and PTPRO have been shown to promote the progression of CRC by reprogramming fatty acid metabolism [12, 13]. Additionally, studies indicate that the upregulation of CPT1A mediated by ALKBH5 enhances fatty acid metabolism in macrophages and promotes M2 macrophage polarization, thereby facilitating the malignant progression of CRC [14]. LncRNAs have emerged as critical players in tumorigenesis and tumor progression [15–17]. The m6A modification–mediated lncRNA POU6F2-AS1 has been reported to reprogram fatty acid metabolism and promote CRC growth by upregulating FASN [18]. Another study constructed a prognostic model based on fatty acid metabolism-related genes, determining its ability to predict the efficacy of immunotherapy in CRC patients [19]. Consequently, the aforementioned genes have been confirmed as markers of fatty acid metabolism in CRC (Table 1).

### 2.2. The Interaction Mechanism Between Fatty Acid Metabolism and CRC

A complex and bidirectional interaction mechanism is present between fatty acid metabolism and CRC. On one side, the development and advancement of CRC can result in the reprogramming of fatty acid metabolism. To satisfy the needs for quick proliferation and survival, cancer cells increase de novo fatty acid synthesis and uptake while simultaneously suppressing FAO. For instance, studies have shown that the expression of FASN is upregulated in CRC, promoting fatty acid synthesis to provide tumor cells with essential raw materials for biomembrane synthesis and energy, thereby supporting tumor growth and metastasis. On the other hand, alterations in fatty acid metabolism can reciprocally influence CRC. Abnormal fatty acid metabolites can affect the signaling pathways of tumor cells. Certain fatty acids, for example, have the ability to influence the Wnt/*β*-catenin signaling pathway, a key component in the advancement and development of CRC. When this pathway is activated improperly, it can lead to increased tumor cell proliferation and stem-like properties [20]. Additionally, fatty acid metabolism interacts with the tumor microenvironment. Immune and stromal cells within this environment can influence tumor progression by modulating fatty acid metabolism. For example, tumor-associated macrophages (TAMs) can undergo M2 polarization under the influence of fatty acid metabolism. These polarized TAMs can secrete various cytokines that promote tumor cell proliferation, angiogenesis, and immune escape, thereby driving the development of CRC [14].

### 2.3. Fatty Acid Metabolites as Diagnostic Markers for CRC

Fatty acid metabolites hold significant potential for application in the diagnosis of CRC. Studies have demonstrated that the levels of specific fatty acid metabolites in patients with CRC differ markedly from those in healthy individuals. For instance, serum metabolomic analyses comparing CRC patients to healthy controls have revealed significant alterations in certain acylcarnitines, such as C6DC and C4-OH, in the serum of patients. These alterations can effectively distinguish CRC patients from healthy individuals, achieving a high diagnostic efficacy with an area under the receiver operating characteristic curve (AUC) of 0.837 [21]. Furthermore, the levels of SCFAs are notably reduced in patients with CRC. SCFAs are produced through the fermentation of dietary fiber by gut microbiota and possess various physiological functions. A decrease in SCFA levels may indicate dysbiosis of the gut microbiota and abnormalities in fatty acid metabolism. Research has shown that supplementation with SCFA-producing probiotics can exert an inhibitory effect on CRC, suggesting that SCFAs or their related metabolites may serve as potential biomarkers for the diagnosis and therapeutic monitoring of CRC [10]. Additionally, metabolites of long-chain polyunsaturated fatty acids, such as prostaglandin E2 derived from arachidonic acid metabolism, are associated with the development and progression of CRC. Changes in their levels can serve as indicators for assessing tumor progression [22].

## 3. Fatty Acid Metabolism and CRC Treatment

### 3.1. Drugs Targeting Fatty Acid Metabolism for CRC Treatment

The development of therapeutic drugs targeting the characteristics of fatty acid metabolism has provided new directions for the treatment of CRC. Certain drugs exert their effects by inhibiting key enzymes involved in fatty acid synthesis, such as the FASN inhibitor orlistat, which has been shown to inhibit CRC growth and enhance sensitivity to chemotherapeutic agents in in vitro cell experiments and animal models. Studies indicate that FASN is highly expressed in CRC tissues and is associated with tumor proliferation and metastasis. Inhibiting FASN reduces fatty acid synthesis, impacting the energy supply and membrane structure synthesis of tumor cells, thereby inhibiting tumor growth [23]. Additionally, drugs targeting the FAO process are under investigation. For instance, by inhibiting Carnitine Palmitoyltransferase 1A (CPT1A), the entry of fatty acids into mitochondria for oxidation can be obstructed, thus suppressing the energy metabolism of tumor cells. Some studies have demonstrated that CPT1A inhibitors possess therapeutic potential for CRC, inducing apoptosis in tumor cells and inhibiting tumor progression [12]. Meanwhile, certain natural products and their derivatives have also been identified to regulate fatty acid metabolism and inhibit CRC. For example, components derived from traditional Chinese medicine can exert antitumor effects by modulating signaling pathways related to fatty acid metabolism, thereby providing additional therapeutic options for the treatment of CRC [24].

### 3.2. Fatty Acid Metabolism and Chemotherapy for CRC

The management of fatty acid metabolism is crucial in the treatment of CRC through chemotherapy. Although chemotherapeutic drugs are effective in eliminating tumor cells, these cells can adjust to the pressures exerted by chemotherapy by modifying their fatty acid metabolism, which leads to the development of resistance to the drugs. For example, research has shown that fatty acid synthesis increases in CRC cells that are resistant to oxaliplatin and the expression levels of FASN are also elevated. Targeting FASN can potentially reverse the resistance to oxaliplatin and improve the effectiveness of chemotherapy. This effect may be associated with the modulation of the MAPK/ERK and PI3K/AKT signaling pathways, which are essential for processes such as tumor cell growth, programmed cell death, and resistance to drugs [23]. Moreover, the regulation of fatty acid metabolism can also influence the efficacy of chemotherapeutic drugs. Modulating fatty acid metabolism can alter the energy metabolic state of tumor cells, making them more sensitive to chemotherapeutic agents. For instance, certain drugs inhibit FAO, disrupting the energy supply to tumor cells and enhancing the cytotoxic effects of chemotherapeutic agents. Simultaneously, the regulation of fatty acid metabolism can impact the tumor microenvironment and modulate the function of immune cells, indirectly augmenting the efficacy of chemotherapy. For instance, modulating the fatty acid metabolism of TAMs can alter their polarization state, enhancing the ability of immune cells to kill tumor cells and synergizing with chemotherapy to exert antitumor effects [14].

### 3.3. Fatty Acid Metabolism and Immunotherapy for CRC

Integrating fatty acid metabolism with CRC immunotherapy represents an emerging therapeutic strategy. The fatty acid metabolism of tumor cells can influence the function of immune cells and the immune status of the tumor microenvironment. For instance, the expression of FASN can inhibit the activity of tumor-infiltrating immune cells, leading to tumor immune escape. Inhibiting FASN can enhance the sensitivity of tumor cells to immune cells, thereby improving the efficacy of immunotherapy [25]. Moreover, various metabolites of fatty acids possess the ability to influence the polarization and function of immune cells. For instance, SCFAs can modulate the activity of immune cells like T cells and macrophages, leading to immune activation and strengthened antitumor responses. In individuals diagnosed with CRC, increasing SCFA production through supplementation or alteration of gut microbiota can enhance the immune environment of the tumor and complement immunotherapy treatments. At the same time, the strategy of combining immune checkpoint inhibitors with approaches that target fatty acid metabolism pathways appears to offer promising therapeutic opportunities. For example, in CRC characterized by ARID1A deficiency, blocking arachidonic acid metabolism may improve the effectiveness of immune checkpoint inhibitors by stimulating CD8+ T-cell activation and inhibiting vasculogenic mimicry, thereby reinforcing the tumoricidal effects mediated by the immune system [26].

## 4. Fatty Acid Metabolism and CRC Treatment Resistance

Fatty acid metabolism plays a significant role in chemotherapy resistance in CRC. Studies have demonstrated that CRC cells resistant to chemotherapy frequently exhibit alterations in fatty acid metabolism, including increased fatty acid synthesis and abnormal FAO. Taking oxaliplatin resistance as an example, the expression of FASN is upregulated in resistant cells, promoting de novo fatty acid synthesis. This process provides additional energy and raw materials for biomembrane synthesis, enabling tumor cells to continuously proliferate and survive under the pressure of chemotherapeutic agents [23]. Additionally, fatty acid metabolism can mediate chemotherapy resistance by influencing intracellular signaling pathways. For instance, abnormal fatty acid metabolism can activate the PI3K/AKT/mTOR signaling pathway, which promotes tumor cell growth and survival, as well as the expression of drug resistance-related proteins, thereby leading to chemotherapy resistance. Furthermore, fatty acid metabolites, particularly certain unsaturated fatty acids, can regulate the fluidity and permeability of cell membranes. This regulation affects the uptake and distribution of chemotherapeutic drugs, thereby reducing their effective concentration within tumor cells and ultimately contributing to the development of drug resistance [27]. The fatty acid metabolic pathways associated with drug resistance in CRC primarily encompass the synthesis, uptake, and oxidation of fatty acids. Within the fatty acid synthesis pathway, aberrant expression of key enzymes such as acetyl-CoA carboxylase (ACC) and FASN is closely linked to chemotherapy resistance. Studies indicate that in drug-resistant cells, the activities of ACC and FASN are enhanced, facilitating fatty acid synthesis and providing survival advantages to tumor cells [23]. In the fatty acid uptake pathway, proteins such as CD36 and fatty acid–binding proteins (FABPs) play a crucial role in the transmembrane transport of fatty acids. Upregulation of these proteins can increase fatty acid uptake by tumor cells, thereby meeting their metabolic demands during chemotherapy stress and promoting drug resistance. For instance, in certain drug-resistant CRC cell lines, elevated expression of CD36 allows these cells to absorb more fatty acids, sustaining their growth and survival [28]. In the FAO pathway, alterations in the activity of enzymes such as CPT1A are also implicated in drug resistance. Inhibition of CPT1A can impair FAO, leading to the accumulation of fatty acids within cells and disrupting cellular energy metabolism and signal transduction, thereby fostering the development of drug resistance [12].

Overcoming chemotherapy resistance in CRC through regulatory strategies targeting fatty acid metabolism is of paramount significance. Inhibiting key enzymes involved in fatty acid synthesis can reverse resistance; for instance, FASN inhibitors reduce fatty acid synthesis, thereby decreasing the energy supply and membrane synthesis materials for tumor cells, which enhances their sensitivity to chemotherapy drugs. Preclinical studies indicate that the combination of FASN inhibitors with chemotherapy drugs significantly improves chemotherapy efficacy and inhibits tumor growth [23]. Furthermore, modulating FAO emerges as another viable strategy to overcome resistance. By activating FAO, the energy consumption of tumor cells increases, consequently reducing their tolerance to chemotherapy drugs. For example, PPAR*α* agonists can upregulate the expression of CPT1A, promote FAO, and enhance the cytotoxicity of chemotherapeutic agents. Additionally, modifying the gut microbiota to influence fatty acid metabolism presents a promising strategy; supplementing with probiotics or dietary fibers to boost the production of SCFAs can regulate the tumor microenvironment, inhibit tumor cell growth, and mitigate drug resistance, thereby synergizing with chemotherapy [10]. Concurrently, targeted therapies that focus on signaling pathways related to fatty acid metabolism, such as the inhibition of the PI3K/AKT/mTOR signaling pathway, have shown effectiveness in overcoming chemotherapy resistance in CRC.

## 5. The Role of Fatty Acid Metabolism in the Immune Microenvironment of CRC

The metabolism of fatty acids is essential in influencing the immune microenvironment associated with CRC. Studies suggest that irregularities in fatty acid metabolism are significantly connected to the initiation and advancement of CRC. These fatty acids not only act as a source of energy for cancerous cells but also affect the tumor microenvironment by altering cell signaling and immune responses [5]. In relation to CRC, the metabolism of fatty acids is closely linked to the roles of immune cells, especially the polarization and functioning of TAMs [29]. TAMs facilitate tumor growth and immune evasion by reprogramming fatty acid metabolism [30]. In addition, the metabolism of fatty acids plays a role in the immune reaction to therapy in CRC. Research has shown that the activity of genes linked to fatty acid metabolism may act as indicators for how CRC patients respond to immunotherapy [31]. Modifying fatty acid metabolism can either boost or suppress the antitumor functions of immune cells through the regulation of their metabolic conditions, which ultimately impacts the effectiveness of immunotherapy [32]. In the CRC tumor microenvironment, the interaction between fatty acid metabolism and gut microbiota plays a crucial role. The metabolites produced by gut microbiota, especially SCFAs, affect the immune microenvironment of tumors by modulating immune cell functions and inflammatory responses [10]. The development and progression of CRC is strongly associated with the levels of SCFAs, and it has been demonstrated that supplementing with SCFAs or introducing bacteria that produce them can hinder tumor growth [33]. In summary, the metabolism of fatty acids plays various roles within the immune microenvironment of CRC, influencing not only the proliferation and spread of tumor cells but also the effectiveness of immunotherapy through the modulation of immune cells' functions and their metabolic conditions [34]. This insight offers new perspectives and potential therapeutic targets for the treatment of CRC.

## 6. Conclusion

CRC, a highly prevalent malignant tumor of the digestive system worldwide, has its occurrence, progression, and mechanisms of drug resistance closely linked to abnormal fatty acid metabolism. This review systematically summarizes the critical role of fatty acid metabolism in the initiation, progression, and drug resistance of CRC, emphasizing its significance as a metabolic marker and a potential therapeutic target in cancer treatment. In-depth research into these mechanisms not only enhances our understanding of the disease but also opens new avenues for precision diagnosis and treatment. Moving forward, prioritizing the exploration of the regulatory network of fatty acid metabolism will facilitate its translation into effective therapeutic strategies in clinical applications.

## Figures and Tables

**Table 1:** Research progress on genes related to fatty acid metabolism.

**Genes**	**Mechanism of action**	**Function**
FUT2	FUT2 promotes YAP1 nuclear translocation and stabilizes mSREBP-1 by fucosylation	Reprogramming fatty acid metabolism
SDHC	SDHC silencing leads to lipid accumulation through the activation of the PI3K/AKT signaling axis	Reprogramming fatty acid metabolism
ALKBH5	ALKBH5 enhances fatty acid metabolism and M2 polarization of macrophages by upregulating CPT1	Reprogramming fatty acid metabolism
POU6F2-AS1	METTL3-induced m6A modification is involved in the upregulation of POU6F2-AS1	Reprogramming fatty acid metabolism
PTPRO	PTPRO attenuation decreased the fatty acid oxidation rate by repressing the expression of PPAR*α* and its downstream enzyme peroxisomal ACOX1 via activating the p38/ERK MAPK signaling pathway	Reprogramming fatty acid metabolism

## Data Availability

The data that support the findings of this study are available from the corresponding authors upon reasonable request.
